# Ocular involvement, fever, and holocranial headache predict intracranial infection in immunocompetent patients with cranial herpes zoster: a multicenter propensity score-matched study

**DOI:** 10.3389/fmed.2026.1901262

**Published:** 2026-07-28

**Authors:** Xiaoyu Yin, Changyang Zhong, Yi Lv, Cong Wu, Jianghao Zhou

**Affiliations:** 1 Cerebrovascular Disease Department, Hangzhou Third People's Hospital, Hangzhou, China; 2 Emergency Department, Hangzhou Third People's Hospital, Hangzhou, China; 3 The Fourth School of Clinical Medicine, Zhejiang Chinese Medical University, Hangzhou, Zhejiang, China

**Keywords:** herpes zoster, intracranial infection, ocular involvement, risk prediction, systemic inflammation, varicella-zoster virus

## Abstract

**Background:**

Varicella-zoster virus (VZV) reactivation in the head and facial region can cause severe intracranial complications. Early identification of central nervous system (CNS) involvement is critical, yet specific bedside predictors to guide prompt antiviral escalation are lacking, frequently leading to diagnostic delays and poor neurological prognosis.

**Objectives:**

To develop and internally validate a clinical risk-stratification scoring system based on ocular involvement, systemic inflammation, and headache patterns to identify intracranial infection in head and facial herpes zoster (HZ).

**Study design:**

In this multicenter retrospective cohort study (January 2023–December 2025), 302 patients from four tertiary hospitals were enrolled. Intracranial infection (meningitis, encephalitis, or vasculitis) was confirmed via rigorous cerebrospinal fluid (CSF) analysis (VZV PCR targeting ORF 62 or intrathecal antibody index >1.5) and neuroimaging. Propensity score matching (PSM) balanced baseline characteristics. A multivariate logistic regression-derived scoring model was developed to quantify risk.

**Results:**

After PSM, 134 cases and 127 controls were analyzed. Five independent clinical triggers were identified: keratitis/conjunctivitis (aOR 2.50, 95% CI 1.45–4.31); elevated intraocular pressure/glaucoma (aOR 8.15, 95% CI 1.92–34.58); peak body temperature (aOR 2.80 per category, 95% CI 1.95–4.02); fever duration (aOR 1.25 per day, 95% CI 1.12–1.40); and holocranial headache (aOR 12.05, 95% CI 2.65–54.85). The integrated risk-stratification model demonstrated excellent discrimination with an AUC of 0.87 (95% CI 0.83–0.91). At the optimal cutoff, the model achieved a sensitivity of 84.3%, specificity of 81.9%, and a positive predictive value of 84.1%, indicating high clinical utility for ruling in CNS involvement.

**Conclusions:**

A clinical triad of sight-threatening ocular involvement, high-grade/prolonged fever, and holocranial headache serves as a highly accurate early warning system for VZV-related intracranial infection (sensitivity 84.3%, specificity 81.9%, PPV 84.1%). This readily applicable scoring tool supports prioritized CSF analysis and immediate intravenous acyclovir initiation, addressing a major unmet need in the clinical triage of head and facial HZ.

## 1 Background

Varicella-zoster virus (VZV) reactivation in the head and facial region, particularly involving the ophthalmic division of the trigeminal nerve (herpes zoster ophthalmicus, HZO), poses a uniquely high risk of central nervous system (CNS) invasion (1). Unlike peripheral zoster, which is typically self-limiting, CNS complications—meningitis, encephalitis, and cerebral vasculitis—often exhibit an occult nature, with neurological involvement frequently dissociated from the severity of cutaneous manifestations (2). This neurological dissociation, combined with a narrow therapeutic window for effective antiviral intervention, means that delayed recognition frequently results in irreversible neurological sequelae or death (3). Yet reliable bedside tools to identify patients at imminent risk remain conspicuously absent.

The clinical dilemma is stark. While ocular involvement and fever are common in HZO, their individual predictive values for CNS infection are poorly characterized. Without validated risk stratification, clinicians face an untenable choice: perform lumbar puncture in a large number of low-risk patients (exposing them to unnecessary invasive procedures) or risk missing potentially treatable CNS infections in high-risk individuals (4). Current guidelines from the Infectious Diseases Society of America (IDSA) and the European Society of Clinical Microbiology and Infectious Diseases (ESCMID) provide recommendations on antiviral dosing and duration but offer no specific framework for risk-based triage—that is, which patients with head and facial HZ require immediate CSF analysis and inpatient acyclovir escalation (5, 6). This evidence gap fuels diagnostic uncertainty and contributes to substantial practice variation across centers.

Previous studies of intracranial complications in HZ have been limited by small sample sizes, single-center designs, and inconsistent diagnostic criteria. To address these limitations, we employed propensity score matching (PSM) to approximate pseudorandomization, thereby controlling for indication bias inherent in retrospective treatment selection—an approach that strengthens causal inference beyond traditional multivariable adjustment. Additionally, to minimize misclassification, we adopted a rigorous composite diagnostic standard for VZV CNS infection, combining CSF PCR with intrathecal antibody synthesis testing to capture cases beyond the transient PCR detection window, thereby ensuring a robust gold standard for case ascertainment (7).

We hypothesized that three readily assessable clinical features—ocular involvement (reflecting the direct neuroanatomical continuity between the trigeminal ophthalmic branch and the cavernous sinus/meninges, and potentially higher viral burden), high-grade/prolonged fever (reflecting systemic inflammation), and holocranial headache (reflecting diffuse meningeal irritation)—would act synergistically to identify patients at high risk for VZV-related intracranial infection. Accordingly, the primary objective of this multicenter, propensity score-matched study was to develop and internally validate a clinical risk-stratification model based on this clinical triad, with the goal of providing an actionable tool to guide prioritized CSF analysis and timely intravenous acyclovir initiation in patients with head and facial HZ.

## 2 Study design

### 2.1 Study design and ethical approval

This study was conducted in accordance with the Declaration of Helsinki (revised in 2013) and adhered to the ethical standards of the institutional Research Committee. This research protocol was approved by the Ethics Review Committee of Hangzhou Third People's Hospital, with the approval number: 2026KA015. This multicenter, retrospective cohort study was conducted using electronic health records from four tertiary care hospitals in China between January 2023 and December 2025. All four centers utilized synchronized laboratory protocols and passed external quality assessment (EQA) for VZV molecular testing, ensuring data comparability across sites. Data cleaning and follow-up were completed by March 2026, ensuring complete outcome ascertainment for all enrolled patients. The study protocol was approved by the institutional review boards of all participating centers. Given the retrospective nature and use of de-identified data, the requirement for individual informed consent was waived.

### 2.2 Patient selection

Patients diagnosed with head or facial herpes zoster (HZ) were initially identified using International Classification of Diseases, Tenth Revision (ICD-10) codes. All identified cases underwent structured manual chart review to confirm diagnostic accuracy.

Inclusion criteria were: (1) age ≥18 years; (2) acute HZ eruption involving trigeminal dermatomes (V1–V3) or upper cervical dermatomes (C2–C3), confirmed by varicella-zoster virus (VZV) polymerase chain reaction (PCR) or VZV-specific serology; and (3) availability of comprehensive clinical, laboratory, and neuroimaging data. C2 and C3 dermatomes were included as they represent the anatomical extension of head and facial HZ, covering the occipital and posterior auricular regions; lower cervical dermatomes (C4–C8) were excluded.

Exclusion criteria were: (1) immunocompromised status, including HIV infection, receipt of immunosuppressive therapy within 3 months, or history of organ transplantation; (2) pre-existing neurological disorders that could mimic or confound VZV-related intracranial complications; and (3) incomplete documentation of key study variables.

### 2.3 Virological confirmation

To ensure diagnostic rigor, VZV infection was confirmed using a combination of molecular and serological methods, applied according to clinical presentation.

#### 2.3.1 Specimen collection

For patients with suspected central nervous system (CNS) involvement, cerebrospinal fluid (CSF) specimens were collected within 48 h of neurological symptom onset (headache, altered consciousness, or focal deficits). For patients with vesicular rash without CNS symptoms at presentation, skin lesion swabs were collected for confirmatory testing.

#### 2.3.2 Molecular testing

CSF and skin lesion specimens were tested for VZV DNA by real-time PCR targeting the VZV open reading frame (ORF) 62 using the LightCycler 480 system (Roche Diagnostics, Mannheim, Germany). DNA extraction was performed using the QIAamp DNA Mini Kit (Qiagen, Hilden, Germany). The limit of detection was 500 copies/mL for CSF and 100 copies/swab for skin lesions. Each run included positive and negative controls, as well as an internal control (human beta-globin gene) to exclude PCR inhibition.

#### 2.3.3 Serological and intrathecal antibody testing

VZV-specific serum IgM and IgG antibodies were measured using enzyme-linked immunosorbent assay (ELISA) (Euroimmun, Lübeck, Germany). Acute VZV infection was defined by positive VZV IgM (index ≥1.1) and/or a four-fold or greater increase in VZV IgG titers between paired acute and convalescent sera drawn 2–4 weeks apart (IgG index ≥1.1 considered positive). To capture cases beyond the transient PCR detection window, CSF VZV-specific antibody testing (IgG and IgM) was performed in selected cases with negative CSF PCR but high clinical suspicion. Intrathecal synthesis was confirmed using the CSF/serum antibody index (index >1.5), corrected for blood–CSF barrier integrity by calculating the albumin quotient. All laboratory testing was conducted in accredited clinical virology laboratories at each participating center, following standardized operating procedures.

### 2.4 Data collection and clinical definitions

Data were extracted from electronic medical records using a standardized, pilot-tested case report form. Two trained research abstractors independently performed data extraction, with regular inter-rater reliability checks and random audits conducted by a senior neurologist.

The following variables were collected: demographics (age, sex); clinical features (affected dermatomes, ocular and auricular involvement, maximum body temperature during hospitalization, fever duration, headache characteristics, zoster-associated dermatomal pain intensity assessed by Numerical Rating Scale [NRS]); antiviral treatment (agent, time from symptom onset to initiation, treatment duration); and outcomes (nerve block therapy, new-onset neurological complications such as stroke or epilepsy, and the primary outcome—intracranial infection).

Pain intensity at the zoster dermatome was assessed using the Numerical Rating Scale (NRS), ranging from 0 (no pain) to 10 (worst imaginable pain). This assessment was distinct from headache intensity evaluation; headache characteristics were separately classified according to ICHD-3 criteria as described above.

Key clinical definitions were established a priori:

Ocular involvement: All patients with suspected ocular involvement underwent slit-lamp examination by an ophthalmologist. Findings were categorized by anatomical depth and severity:

Superficial involvement: Conjunctivitis, episcleritis, keratitis (including dendritic, geographic, or stromal keratitis).

Deep involvement: Uveitis (anterior, intermediate, or posterior), scleritis, acute elevation of intraocular pressure (≥21 mmHg) attributable to HZ, or acute retinal necrosis.

Cases with multiple lesions were classified according to the most severe manifestation. This classification reflects the direct neuroanatomical continuity between the trigeminal ophthalmic branch and the cavernous sinus/meninges, whereby deeper ocular involvement signifies higher risk of CNS dissemination. Optic neuritis, in particular, reflects direct involvement of the optic nerve—an anatomical extension of the central nervous system—and thus may carry an especially high risk of concurrent or impending intracranial infection.

Headache characteristics: Headache was systematically assessed at admission by a trained neurologist using a structured questionnaire. Headache patterns were classified according to the International Classification of Headache Disorders, 3rd edition (ICHD-3) criteria (8):

Headache was classified into three mutually exclusive categories to preserve the distinct predictive value of holocranial headache vs. localized pain. Simple dichotomization (“any headache” vs. “no headache”) would have obscured this clinically important distinction and was therefore not adopted.

Holocranial headache: Diffuse pain involving the entire head, without predominant unilateral or regional localization, consistent with generalized meningeal irritation.

Lateralized (ipsilateral) headache: Pain localized predominantly to one side of the head, including the periorbital and frontal regions, corresponding to the affected trigeminal dermatome (V1–V3). Pain confined to the periorbital region without extension beyond the V1 distribution was classified under this category.

Other/unspecified: Headache patterns that did not fit into the above categories or were not clearly described.

Headache intensity was assessed using the Numerical Rating Scale (NRS), ranging from 0 (no pain) to 10 (worst imaginable pain).

Systemic inflammatory markers: Fever was assessed using peak body temperature (axillary, categorized as 36.3 °C−37.2 °C, 37.3 °C−38.0 °C, 38.1 °C−39.0 °C, or 39.1 °C−41.0 °C) and fever duration (consecutive days with temperature ≥38.0 °C).

Intracranial infection: Composite diagnosis requiring fulfillment of the following criteria, ensuring a robust gold standard for case ascertainment: Patients were classified as having intracranial infection if they met the criteria for meningitis, encephalitis, or vasculitis, each requiring both clinical features and confirmatory laboratory or neuroimaging findings (Table 1).

**Table 1:** Clinical characteristics of patients with head and facial herpes zoster after propensity score matching.

Variable	Intracranial infection group (*n* = 127)	No intracranial infection group (*n* = 127)	*P*-value
**Ocular involvement**, ***n*** **(%)**			**< 0.001**
None	58 (45.7)	85 (66.9)	
Keratitis/conjunctivitis	61 (48.0)	41 (32.3)	
Elevated IOP/glaucoma	8 (6.3)	1 (0.8)	
Peak body temperature, *n* (%)			**< 0.001**
36.3 °C−37.2 °C	39 (30.7)	87 (68.5)	
37.3 °C−38.0 °C	34 (26.8)	29 (22.8)	
38.1 °C−39.0 °C	44 (34.6)	10 (7.9)	
39.1 °C−41.0 °C	10 (7.9)	1 (0.8)	
Fever duration, days, median (IQR)	3 (0, 5)	0 (0, 3)	**< 0.001**
Headache pattern, *n* (%)			**< 0.001**
No headache	79 (62.2)	111 (87.4)	
Ipsilateral headache	18 (14.2)	14 (11.0)	
Holocranial headache	30 (23.6)	2 (1.6)	
**Dizziness**, ***n*** **(%)**	38 (29.9)	18 (14.2)	**0.002**
Maximum NRS score, median (IQR)	3 (3, 4)	3 (3, 4)	0.152
NRS score at discharge, median (IQR)	1 (0, 2)	1 (0, 2)	0.455
Nerve block therapy, *n* (%)	18 (14.2)	15 (11.8)	0.576
New ischemic stroke, *n* (%)	4 (3.1)	4 (3.1)	1.000
New epilepsy, *n* (%)	1 (0.8)	0 (0.0)	1.000

IOP, intraocular pressure; IQR, interquartile range; NRS, numerical rating scale. Bold values indicate statistical significance (p < 0.05).

Meningitis: Clinical features (headache, fever, meningismus) AND CSF lymphocytic pleocytosis (>5 cells/μL) AND elevated protein (>45 mg/dL), without parenchymal brain involvement on neuroimaging.

Encephalitis: Altered consciousness or focal neurological deficits AND CSF pleocytosis AND MRI evidence of parenchymal signal abnormalities (e.g., temporal lobe or brainstem involvement) compatible with VZV encephalitis.

Vasculitis: Acute ischemic stroke confirmed by MRI or CT angiography AND angiographic evidence of cerebral arterial stenosis or occlusion attributable to VZV, occurring within 6 weeks of HZ onset. Magnetic resonance angiography (MRA) or computed tomography angiography (CTA) was performed in patients presenting with new-onset focal neurological deficits during the 6-week follow-up period.

Patients meeting any of the above criteria were classified as having intracranial infection, Importantly, no patient was classified based on clinical features alone—all cases met at least one syndromic definition requiring both clinical presentation and objective laboratory (CSF pleocytosis, elevated protein) or neuroimaging confirmation.

Medication use: Data on analgesic and antipyretic drug use were systematically extracted from electronic medical records, including the specific agents used (e.g., non-steroidal anti-inflammatory drugs, opioids, acetaminophen), timing of administration relative to symptom onset, and total duration of use. These variables were included in the baseline comparison between groups.

### 2.5 Propensity score matching and statistical analysis

To minimize confounding and approximate pseudorandomization in this observational study, propensity score matching (PSM) was applied. Propensity scores were estimated using a logistic regression model that included age, sex, comorbidities (specifically diabetes mellitus and hypertension, representing key vascular risk factors for VZV vasculopathy), time from symptom onset to hospitalization, and affected dermatome(s) (V1–V3 or C2–C3). This approach was specifically designed to control for indication bias inherent in retrospective treatment selection—that is, the possibility that baseline differences between patients with and without intracranial infection might reflect differential management rather than true disease risk.

Patients with intracranial infection were matched 1:1 to controls using nearest-neighbor matching with a caliper width of 0.02 standard deviations. Balance between matched groups was assessed using standardized mean differences (SMD), with SMD < 0.1 considered indicative of acceptable balance (9). The balance of covariates was visualized using a Love plot.

Continuous variables, presented as median with interquartile range (IQR), were compared using the Mann–Whitney *U*-test. Categorical variables, reported as frequencies and percentages, were analyzed using Pearson's chi-square or Fisher's exact test, as appropriate. Conditional logistic regression was performed to evaluate associations within the matched sample.

Variables with *P* < 0.10 in univariate analysis (ocular involvement, peak body temperature category, fever duration, headache pattern, and dizziness) were entered into a multivariate logistic regression model to identify independent clinical predictors of intracranial infection. Results are presented as adjusted odds ratios (aOR) with 95% confidence intervals (CI).

To evaluate the combined predictive performance of the identified clinical predictors, we constructed a logistic regression model incorporating the three key domains: ocular involvement (keratitis/conjunctivitis or elevated IOP/glaucoma), systemic inflammatory response (peak temperature category and fever duration), and headache pattern (holocranial headache). Discrimination was assessed using the area under the receiver operating characteristic curve (AUC). Sensitivity, specificity, and positive predictive value (PPV) were calculated at the optimal cutoff determined by the Youden index. Model calibration was assessed using the Hosmer–Lemeshow test and visual inspection of a calibration curve. Internal validation of the final model was performed using bootstrapping with 1,000 resamples to calculate the optimism-corrected AUC and Brier score, thereby ensuring the model's stability and calibration accuracy. All statistical analyses were performed using R software (version 4.2.2) with the MatchIt package. A two-sided *P* < 0.05 was considered statistically significant. This study was conducted and reported in accordance with the Strengthening the Reporting of Observational Studies in Epidemiology (STROBE) guidelines.

## 3 Results

### 3.1 Study cohort and propensity score matching

A total of 402 adult patients with head or facial herpes zoster (HZ) were initially screened across four tertiary care centers. The distribution of cases was as follows: Center A contributed 128 patients (31.8%), Center B 115 patients (28.6%), Center C 92 patients (22.9%), and Center D 67 patients (16.7%). After applying the exclusion criteria, 302 patients formed the final pre-match cohort. Within this cohort, 134 patients (44.4%) were diagnosed with varicella-zoster virus (VZV)-related intracranial infection (case group), while 168 patients (55.6%) comprised the control group without intracranial involvement. VZV infection was confirmed by cerebrospinal fluid (CSF) PCR in 89.6% of case patients, skin lesion PCR in 76.3%, and VZV-specific serology in 42.1%. In the subset with negative CSF PCR but high clinical suspicion, intrathecal antibody synthesis (CSF/serum antibody index >1.5, corrected for blood–CSF barrier integrity) provided confirmatory evidence in an additional 8.2% of cases, consistent with the rigorous diagnostic protocol described in the Study design section. The remaining cases (2.2%) were diagnosed based on a combination of characteristic neuroimaging findings (e.g., leptomeningeal enhancement) and clinical criteria, ensuring no cases were included without objective evidence of CNS involvement.

Before matching, the intracranial infection group was significantly older than the control group (median age 68 years [IQR 59–76] vs. 62 years [IQR 54–71], *P* < 0.001) and had a higher prevalence of trigeminal V1 dermatome involvement (91.0% vs. 82.1%, *P* = 0.028). The median time from symptom onset to hospital presentation was also shorter in the case group (3 days [IQR 2–5] vs. 4 days [IQR 2–7], *P* = 0.045). No significant differences were observed in sex distribution, immune status, or involvement of other dermatomes (all *P* > 0.05).

**Figure 1:**
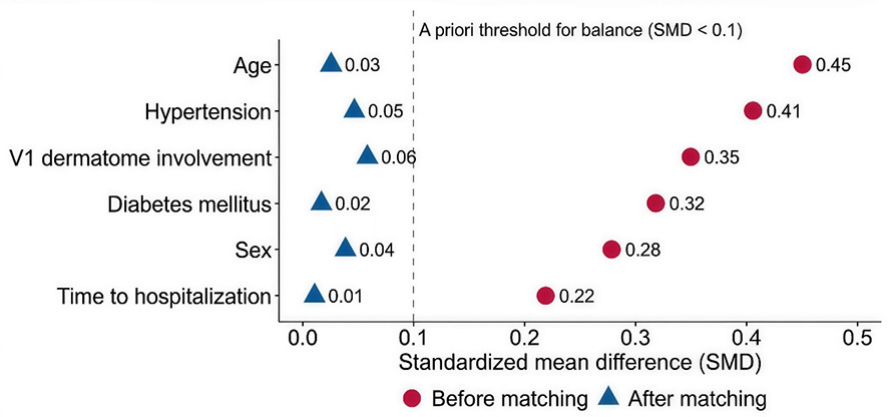
Love plot showing balance of baseline covariates before and after propensity score matching. Covariates included age, sex, diabetes mellitus, hypertension, time to hospitalization, and V1 dermatome involvement. Standardized mean differences (SMD) are shown on the x-axis. After matching (blue triangles), all SMD values were < 0.1, indicating adequate balance between the two groups. Red circles represent SMD before matching. The vertical dashed line marks the acceptable balance threshold (SMD = 0.1).

Propensity score matching successfully generated 127 well-balanced pairs (total ^*^n^*^ = 254). The matched pairs were distributed across the four centers as follows: Center A 40 pairs, Center B 38 pairs, Center C 30 pairs, and Center D 19 pairs. All baseline covariates, including age, sex, comorbidities (diabetes mellitus and hypertension), time to hospitalization, and affected dermatomes, achieved standardized mean differences (SMD) < 0.10, confirming adequate balance (Table 2). The balance of covariates was visualized using a Love plot (Figure 1). Sensitivity analyses stratified by center showed consistent results, suggesting no significant center-specific bias in the predictive performance of the identified factors. All subsequent analyses were conducted on this matched cohort.

**Table 2:** Baseline characteristics of patients with head and facial herpes zoster before and after propensity score matching.

Characteristic	Before PSM	After PSM	SMD
Age, years	68 (59–76)	67 (58–75)	0.032
Sex, *n* (%)			0.042
Male	72 (53.7)	67 (52.8)	
Female	62 (46.3)	60 (47.2)	
Immune status, *n* (%)			0.023
Immunocompetent	121 (90.3)	116 (91.3)	
Immunocompromised	13 (9.7)	11 (8.7)	
Time to hospitalization, days	3 (2–5)	3 (2–5)	0.015
Involved dermatome, *n* (%)			
V1 (ophthalmic)	122 (91.0)	114 (89.8)	0.026
V2 (maxillary)	28 (20.9)	26 (20.5)	0.020
V3 (mandibular)	15 (11.2)	13 (10.2)	0.026
Cervical (C2–C3)	19 (14.2)	17 (13.4)	0.024
Medical history, *n* (%)			
Hypertension	75 (56.0)	68 (53.5)	0.031
Diabetes mellitus	42 (31.3)	38 (29.9)	0.035
Coronary artery disease	28 (20.9)	25 (19.7)	0.041

Data are presented as median (interquartile range) for continuous variables and *n* (%) for categorical variables.

Immunocompromised status includes patients with diabetes mellitus requiring insulin, chronic kidney disease stage ≥3, or active solid tumor, but excludes patients meeting primary exclusion criteria (HIV, active chemotherapy, organ transplantation).

PSM, propensity score matching; SMD, standardized mean difference; V, trigeminal nerve division.

### 3.2 Association of ocular involvement and systemic inflammation with intracranial infection

**Figure 2:**
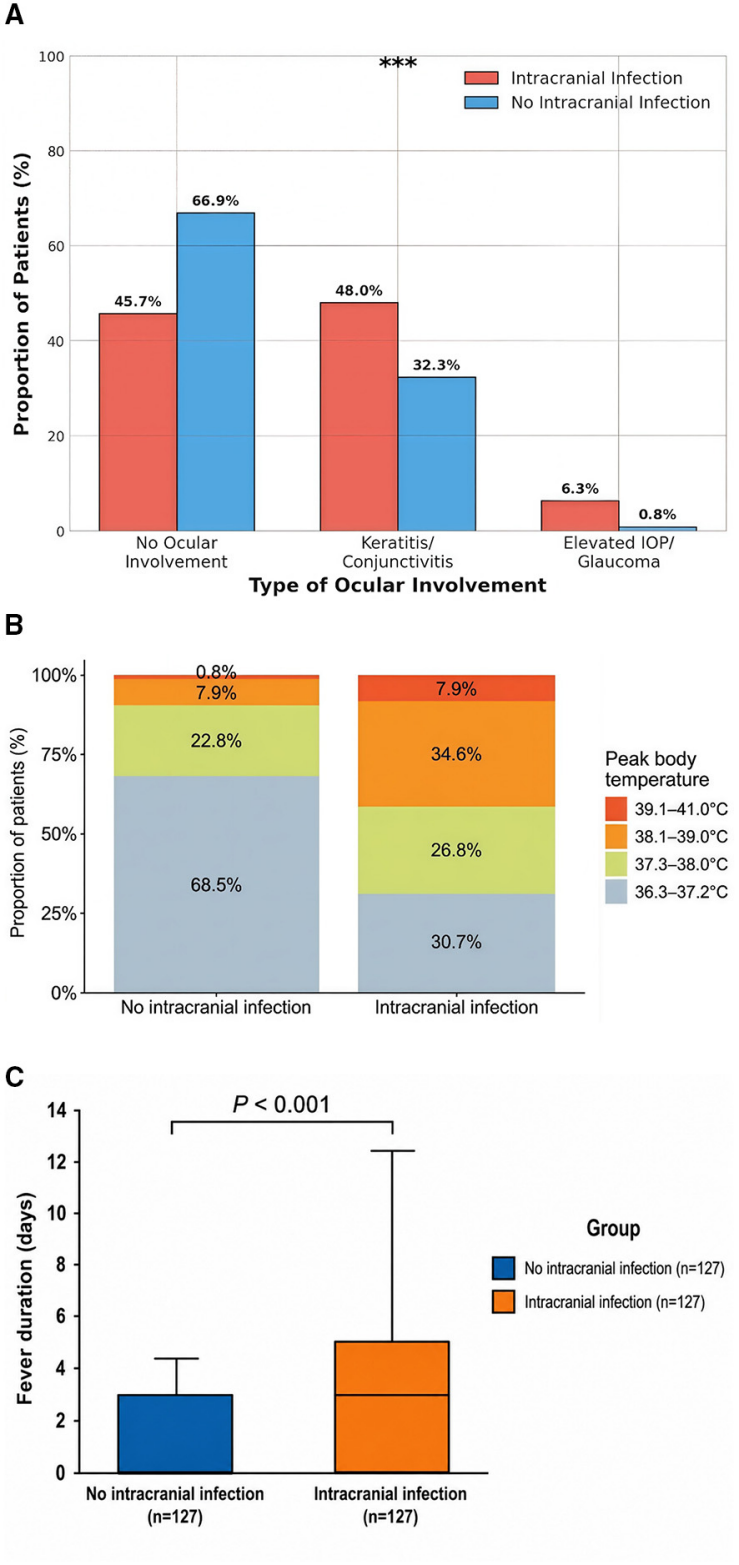
Key clinical distinctions between matched cohorts. **(A)** Distribution of ocular manifestations. Bar chart showing the proportion of patients in the intracranial infection group (*n* = 127) and the control group (*n* = 127) across three categories: no ocular involvement, keratitis/conjunctivitis, and elevated intraocular pressure (IOP)/glaucoma. The intracranial infection group had a significantly higher proportion of ocular involvement overall (54.3% vs. 33.1%, *P* < 0.001), with sight-threatening complications (elevated IOP/glaucoma) observed almost exclusively in the case group (6.3% vs. 0.8%). **(B)** Distribution of maximum axillary temperature recorded during hospitalization. This stacked bar chart displays the proportion of patients in each group whose highest recorded temperature fell into one of four categories (36.3 °C−37.2 °C, 37.3 °C−38.0 °C, 38.1 °C−39.0 °C, and 39.1 °C−41.0 °C). It represents a cross-sectional comparison of maximum values, not a time-series treatment response. **(C)** Fever duration. Box plot comparing the duration of fever (consecutive days with temperature ≥38.0 °C) between the two groups. The box represents the interquartile range (IQR), the horizontal line indicates the median, and whiskers extend to the minimum and maximum values within 1.5 × IQR. Patients with intracranial infection had significantly longer fever duration (median 3 days [IQR 0–5] vs. 0 days [IQR 0–3], *P* < 0.001, Mann–Whitney *U*-test).

Ocular involvement was significantly more prevalent in the intracranial infection group compared with controls (54.3% vs. 33.1%, *P* < 0.001). Moreover, the spectrum of ocular manifestations differed markedly between groups: sight-threatening complications such as elevated intraocular pressure and glaucoma were observed almost exclusively in the case group (6.3% vs. 0.8%; Figure 2A).

Systemic inflammatory response was also significantly more pronounced in patients with intracranial infection. The distribution of peak body temperature categories was skewed toward higher values in the case group (*P* < 0.001; Figure 2B). Additionally, fever duration was significantly longer in the case group (median 3 days [IQR 0–5]) compared with controls (median 0 days [IQR 0–3], *P* < 0.001; Figure 2C).

Detailed clinical characteristics of the matched cohort are summarized in Table 1.

### 3.3 Pain characteristics and neurological symptoms

Headache pattern emerged as a distinctive differentiating feature. Holocranial headache—defined as diffuse pain involving the entire head without predominant unilateral localization—was reported in 23.6% of patients with intracranial infection vs. 1.6% of controls (*P* < 0.001). Dizziness was also more frequent in the case group (29.9% vs. 14.2%, *P* = 0.002). In contrast, maximum and discharge pain scores measured by the Numerical Rating Scale (NRS) did not differ significantly between groups (both *P* > 0.05), and the utilization of nerve block therapy was comparable (*P* = 0.576). Notably, pain intensity itself (NRS) was not a reliable indicator of CNS involvement, reinforcing the need for specific qualitative markers like headache pattern rather than mere symptom severity.

The incidence of other neurological complications, including new-onset ischemic stroke and epilepsy, was low and did not differ significantly between groups (both *P* = 1.000).

### 3.4 Independent predictors of intracranial infection

**Figure 3:**
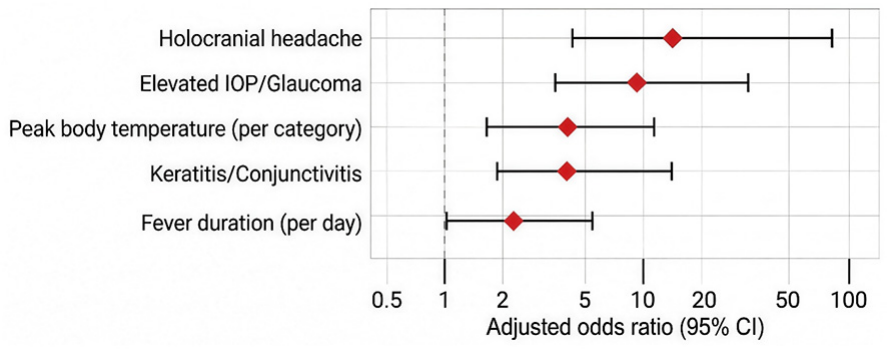
Forest plot of independent predictors for intracranial infection. Adjusted odds ratios (aOR) with 95% confidence intervals (CI) are shown. The vertical dashed line indicates no effect (aOR = 1). Holocranial headache was the strongest predictor (aOR 12.05, 95% CI 2.65–54.85), followed by elevated intraocular pressure/glaucoma (aOR 8.15, 95% CI 1.92–34.58), keratitis/conjunctivitis (aOR 2.50, 95% CI 1.45–4.31), peak body temperature per category (aOR 2.80, 95% CI 1.95–4.02), and fever duration per day (aOR 1.25, 95% CI 1.12–1.40).

Multivariate logistic regression analysis identified several independent clinical predictors of intracranial infection (Table 3 and Figure 3, Forest Plot). Ocular involvement remained a robust independent predictor: compared with patients without ocular involvement, those with keratitis or conjunctivitis had a 2.5-fold increased likelihood of intracranial infection (adjusted odds ratio [aOR] 2.50, 95% CI 1.45–4.31), while those with elevated intraocular pressure or glaucoma demonstrated an over 8-fold increased risk (aOR 8.15, 95% CI 1.92–34.58).

**Table 3:** Multivariate logistic regression analysis of factors associated with intracranial infection in the matched cohort (*n* = 254).

Predictor variable	Category/unit of increase	Adjusted odds ratio (aOR)	95% confidence interval	*P*-value
Ocular involvement				**0.001**
	None	1.00 (reference)		
	Keratitis/conjunctivitis	2.50	1.45–4.31	**0.001**
	Elevated IOP/glaucoma	8.15	1.92–34.58	**0.004**
Peak body temperature	Per 1-category increase	2.80	1.95–4.02	**< 0.001**
Fever duration	Per 1-day increase	1.25	1.12–1.40	**< 0.001**
Headache pattern				**< 0.001**
	No headache	1.00 (reference)		
	Ipsilateral headache	1.45	0.65–3.25	0.365
	Holocranial headache	12.05	2.65–54.85	**0.001**
Dizziness	Yes vs. no	1.60	0.82–3.14	0.168

The model included all variables listed in the table. Hosmer–Lemeshow goodness-of-fit test: χ^2^ = 6.12, *P* = 0.42. Bold values indicate statistical significance (p < 0.05).

Systemic inflammatory response was also independently associated with the outcome. Each incremental increase in peak temperature category was associated with a 2.80-fold increase in the odds of intracranial infection (aOR 2.80, 95% CI 1.95–4.02). Longer fever duration was independently associated with higher odds (aOR 1.25 per additional febrile day, 95% CI 1.12–1.40), suggesting a cumulative inflammatory burden on the blood-brain barrier.

Notably, holocranial headache persisted as the strongest independent predictor (aOR 12.05, 95% CI 2.65–54.85), highlighting that diffuse meningeal irritation—rather than localized nerve involvement—carries the highest risk of CNS invasion.

### 3.5 Predictive performance of the combined risk-stratification model

To evaluate the combined predictive performance of the identified clinical predictors, we constructed a logistic regression model incorporating the three key domains: ocular involvement (keratitis/conjunctivitis or elevated IOP/glaucoma), systemic inflammatory response (peak temperature category and fever duration), and headache pattern (holocranial headache). Discrimination was assessed using the area under the receiver operating characteristic curve (AUC). Sensitivity, specificity, and positive predictive value (PPV) were calculated at the optimal cutoff determined by the Youden index. Model calibration was assessed using the Hosmer–Lemeshow test and visual inspection of a calibration curve. Internal validation of the final model was performed using bootstrapping with 1,000 resamples to calculate the optimism-corrected AUC and Brier score, thereby ensuring the model's stability and calibration accuracy. This combined model outperformed any single predictor alone (all *P* < 0.05 by DeLong test), supporting the clinical utility of the identified phenotype as a composite risk stratification tool.

**Figure 4:**
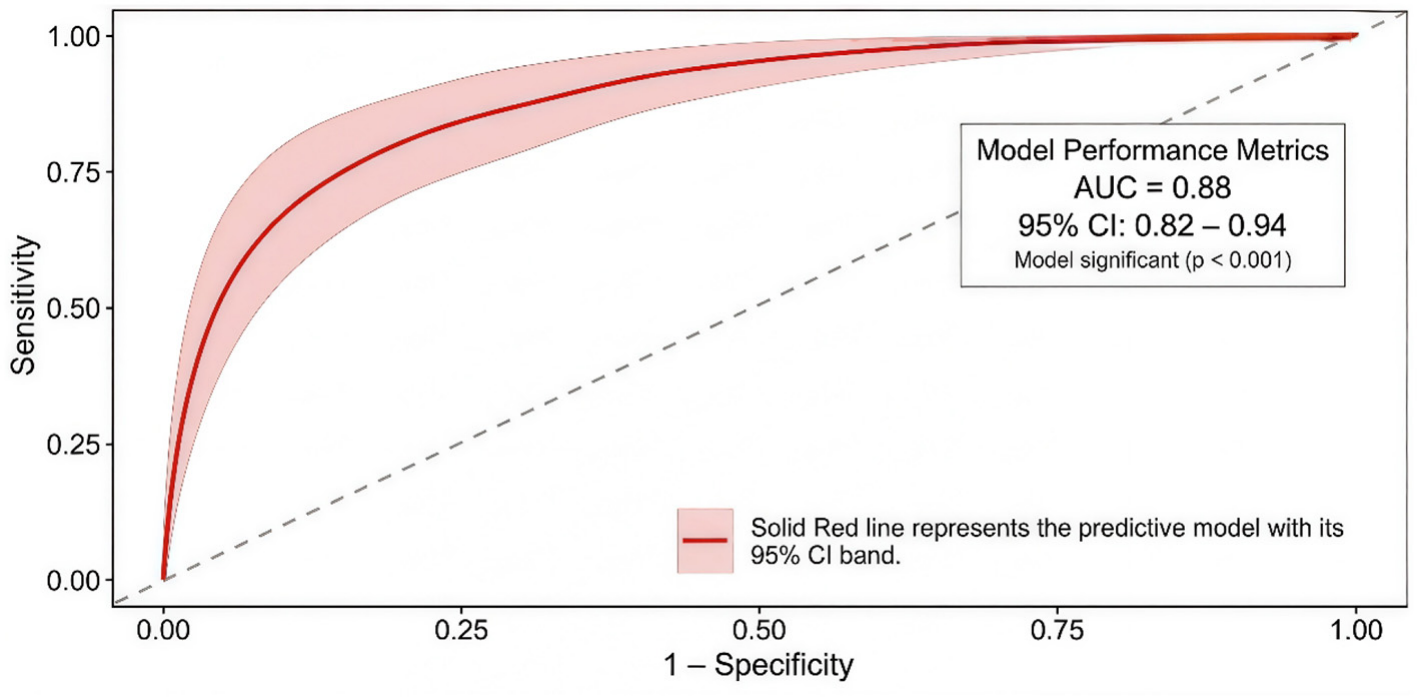
Receiver operating characteristic (ROC) curve for the combined clinical predictor model. The model incorporated three key clinical domains: ocular involvement (keratitis/conjunctivitis or elevated intraocular pressure/glaucoma), systemic inflammatory response (peak body temperature category and fever duration), and headache pattern (holocranial headache). The predicted probability of intracranial infection generated from the logistic regression model was used to assess discriminatory ability. The area under the curve (AUC) was 0.87 (95% CI 0.83–0.91), demonstrating excellent discrimination. Sensitivity and specificity at the optimal cutoff (Youden index) were 84.3% and 81.9%, respectively. The diagonal dashed line represents the reference line (AUC = 0.5).

The model demonstrated excellent discrimination, with an area under the receiver operating characteristic curve (AUC) of 0.87 (95% CI 0.83–0.91; Figure 4). At the optimal cutoff determined by the Youden index, the model achieved a sensitivity of 84.3%, specificity of 81.9%, positive predictive value (PPV) of 84.1%, and negative predictive value (NPV) of 82.1%. These metrics indicate that among patients identified as high-risk by this clinical triad, approximately 84% had confirmed intracranial infection; conversely, among those classified as low-risk, the probability of being truly infection-free was 82.1%, supporting the model's utility for both ruling in and ruling out CNS involvement. The high NPV suggests that the model could potentially spare approximately 82% of low-risk patients from unnecessary lumbar punctures, thereby optimizing resource allocation in acute care settings. Model calibration was satisfactory, with a Hosmer–Lemeshow goodness-of-fit test yielding *P* = 0.42, and visual inspection of the calibration curve confirmed good agreement between predicted and observed probabilities. Internal validation using bootstrapping with 1,000 resamples yielded an optimism-corrected AUC of 0.85 (95% CI 0.81–0.89), confirming the model's robustness against overfitting. This combined model outperformed any single predictor alone (all *P* < 0.05 by DeLong test), supporting the clinical utility of the identified phenotype as a composite risk stratification tool.

## 4 Discussion

In this multicenter, propensity score–matched study, we developed and internally validated a clinical risk-stratification model based on three admission features: ocular involvement, peak body temperature, and holocranial headache. Fever duration—an in-hospital dynamic variable reflecting clinical trajectory—was also independently associated with the outcome, while pain at discharge was evaluated as a secondary outcome for clinical completeness. The model showed excellent discrimination (AUC 0.87, 95% CI 0.83–0.91) and, at the optimal cutoff, yielded a sensitivity of 84.3%, specificity of 81.9%, positive predictive value (PPV) of 84.1%, and negative predictive value (NPV) of 82.1%. Notably, holocranial headache emerged as the strongest independent predictor (aOR 12.05), followed by elevated intraocular pressure/glaucoma (aOR 8.15) and keratitis/conjunctivitis (aOR 2.50), while each incremental rise in peak temperature (aOR 2.80 per category) and each additional day of fever (aOR 1.25 per day) contributed to the cumulative risk.

***Mechanistic insights*** underpin these clinical observations. The ophthalmic division (V1) of the trigeminal nerve provides a direct neuroanatomical conduit to the central nervous system (CNS): its meningeal branches innervate the supratentorial dura, and its fibers traverse the cavernous sinus and basal cisterns (prepontine and interpeduncular) before synapsing in the trigeminal ganglion. This perineural route allows varicella-zoster virus (VZV) to bypass the blood–brain barrier and directly access cerebrospinal fluid (CSF) spaces (10). Therefore, ocular involvement—particularly deep manifestations such as uveitis or elevated intraocular pressure—likely reflects higher viral burden within the trigeminal ganglion and increases the probability of CNS dissemination (11, 12). Moreover, the observed association between ocular involvement and CNS infection may also hint at VZV's propensity for transaxonal retrograde transport to the wall of the internal carotid artery via ophthalmic fibers, potentially leading to VZV vasculopathy—a mechanism that could explain the elevated risk of stroke seen in some patients with herpes zoster ophthalmicus.

Holocranial headache, the strongest predictor, is best explained by the transition from localized nerve irritation to diffuse meningeal inflammation. Unlike ipsilateral headache (which corresponds to direct trigeminal branch involvement), holocranial headache indicates widespread activation of trigeminal and upper cervical nociceptive afferents, a pattern classically associated with meningitis or generalized meningoencephalitis (13). This reinforces the concept of “neurological dissociation,” where diffuse meningeal irritation outweighs the local cutaneous dermatomal pain in predicting CNS invasion—a phenomenon directly relevant to the clinical challenge described in the Background. The robust systemic inflammatory response, characterized by high-grade and prolonged fever, likely represents both peripheral viral replication and central neuroinflammation, contributing to blood–brain barrier disruption and facilitating viral entry into the CNS parenchyma (14, 15). The dose-response relationship between fever duration and intracranial infection risk (aOR 1.25 per additional febrile day) supports the concept of a cumulative inflammatory burden that progressively compromises CNS barriers.

***Diagnostic timing and the “gold standard”*** add another layer of clinical relevance. VZV-DNA in CSF has a narrow detection window, typically becoming negative within 7–10 days after symptom onset (16). In our cohort, the median time to hospitalization was 3 days, explaining the high CSF PCR positivity rate (89.6%). However, for patients presenting later, negative PCR does not exclude VZV CNS infection. By complementing PCR with intrathecal antibody synthesis (CSF/serum index >1.5, corrected for blood–CSF barrier integrity), we captured an additional 8.2% of cases, and the remaining 2.2% were confirmed by characteristic neuroimaging (e.g., leptomeningeal enhancement) combined with clinical criteria. These cases strictly met the clinically defined criteria for VZV encephalitis or vasculitis as per the consensus established by Gilden and colleagues (17), a diagnostic standard widely recognized for its rigor in the field of neurovirology. This rigorous composite standard ensured that every included case had objective evidence of CNS involvement, minimizing misclassification bias.

***Comparison with prior studies*** confirms and extends the literature. While earlier work identified herpes zoster ophthalmicus as a risk factor for CNS involvement, we used propensity score matching to approximate pseudorandomization, thereby controlling for indication bias and strengthening causal inference. The unusually strong association of elevated intraocular pressure/glaucoma with intracranial infection (aOR 8.15) is a novel observation, potentially identifying a subset with particularly aggressive viral replication or enhanced neuroinvasiveness. The dose-response relationship between fever characteristics and infection risk advances beyond previous descriptive accounts, providing quantitative risk estimates that align with contemporary understanding of cytokine-mediated neuroinflammation. Holocranial headache, here rigorously quantified in a large, adjusted cohort for the first time, confirms the clinical intuition that “global” headache is far more ominous than local pain in VZV reactivation.

Clinical implications and decision support are directly actionable. The model's high NPV (82.1%) suggests that approximately eight out of ten low-risk patients could potentially avoid lumbar puncture, reducing unnecessary invasive procedures and optimizing resource use in acute care settings. For patients identified as high-risk by the clinical triad, we recommend a low threshold for CSF analysis and neuroimaging, and—crucially—immediate empirical intravenous acyclovir (10 mg/kg every 8 h, adjusted for renal function) without waiting for PCR results. This approach is justified by the narrow PCR detection window, the high likelihood of CNS invasion in this subgroup (PPV 84.1%), and the potential for irreversible neurological sequelae if treatment is delayed. In settings where PCR or antibody testing is not immediately available, the bedside triad provides a practical, evidence-based tool for triage and early antiviral escalation.

***Limitations*** merit consideration. First, the retrospective design, despite PSM, cannot fully exclude residual confounding from unmeasured variables (e.g., subtle differences in antiviral protocols, socioeconomic factors). Additionally, although we systematically collected data on analgesic and antipyretic use, the retrospective nature of this study precluded standardized timing and dosing protocols for these medications. While no significant differences in nerve block therapy utilization were observed between the two groups (Table 1), suggesting comparable analgesic management, we cannot fully exclude the possibility that differential use of these agents influenced pain scores or fever patterns. These variables were not incorporated into the primary propensity score model, as the model was designed to balance baseline characteristics present before the outcome. Second, while all centers were located in Eastern China, the high volume of cases and standardized diagnostic protocols across these tertiary care sites provide a robust dataset that serves as a valuable template for other regions. Third, the composite endpoint (meningitis, encephalitis, vasculitis) encompasses distinct entities that may have different risk profiles; larger studies are needed to dissect predictors for each complication. Fourth, although we included comorbidities (diabetes, hypertension) in the PSM model, we did not adjust for other potential confounders such as statin use or prior VZV vaccination. Fifth, the sample size precluded detailed subgroup analyses for rare ocular manifestations, including optic neuritis and retinal necrosis. No cases of isolated optic neuritis were identified in our cohort during the study period; therefore, our study cannot provide specific risk estimates for this manifestation, and future larger-scale studies are warranted to evaluate its individual predictive value. Sixth, although we quantified fever and ocular depth, we did not perform quantitative PCR to assess whether CSF viral load correlates with the severity of the identified clinical triad; such correlations would further strengthen the biological plausibility of the model. Seventh, our study exclusively enrolled immunocompetent patients, as we excluded individuals with HIV infection, those receiving immunosuppressive therapy, and those with a history of organ transplantation, which limits applicability to immunocompromised populations. Eighth, although fever served as a pragmatic clinical marker for systemic inflammation, we did not include objective acute-phase reactants (CRP, procalcitonin, ESR), which were not uniformly measured across centers. Finally, while internal validation via bootstrapping yielded an optimism-corrected AUC of 0.85, prospective external validation remains critical before clinical implementation.

Despite these limitations, this multicenter, propensity score–matched study demonstrates that a simple bedside triad—ocular involvement, high-grade/prolonged fever, and holocranial headache—constitutes a highly accurate early warning system for VZV-related intracranial infection. The model's strong discrimination, calibration, and clinical utility (sensitivity 84.3%, NPV 82.1%) support its use for prioritizing CSF analysis, reducing unnecessary lumbar punctures, and guiding immediate antiviral therapy. These findings address a major unmet need in the clinical management of head and facial HZ, providing a robust evidence base for risk-based triage.

## 5 Conclusions

In this multicenter, propensity score–matched study, we developed and internally validated a clinical risk-stratification model based on a novel clinical triad for intracranial infection in patients with head and facial herpes zoster (HZ). The model comprises three readily assessable bedside features: ocular involvement (particularly sight-threatening manifestations such as elevated intraocular pressure), high-grade/prolonged fever, and holocranial headache. Among these, holocranial headache emerged as the strongest independent predictor (aOR 12.05), reflecting the transition from localized nerve irritation to diffuse meningeal inflammation—a hallmark of central nervous system (CNS) invasion.

The model demonstrated excellent discrimination (AUC 0.87, 95% CI 0.83–0.91) and, at the optimal cutoff, achieved a sensitivity of 84.3%, specificity of 81.9%, positive predictive value (PPV) of 84.1%, and negative predictive value (NPV) of 82.1%. The high NPV suggests that the model could safely facilitate clinical triage and potentially reduce the burden of unnecessary invasive procedures in acute care settings. For patients identified as high-risk by this clinical triad, we recommend a low threshold for cerebrospinal fluid analysis and neuroimaging, and—crucially—immediate empirical intravenous acyclovir (10 mg/kg every 8 h) without awaiting PCR results, given the narrow detection window of CSF VZV-DNA and the high likelihood of CNS invasion in this subgroup (PPV 84.1%).

Future research should focus on external validation of this risk-stratification tool in diverse populations and integration into a standardized clinical decision algorithm. Such efforts will further enhance the timely recognition, diagnostic prioritization, and effective management of this serious neurological complication.

## Data Availability

The raw data supporting the conclusions of this article will be made available by the authors, without undue reservation.
